# Slc4 Gene Family in Spotted Sea Bass (*Lateolabrax maculatus*): Structure, Evolution, and Expression Profiling in Response to Alkalinity Stress and Salinity Changes

**DOI:** 10.3390/genes11111271

**Published:** 2020-10-28

**Authors:** Ling-Yu Wang, Yuan Tian, Hai-Shen Wen, Peng Yu, Yang Liu, Xin Qi, Zhi-Chao Gao, Kai-Qiang Zhang, Yun Li

**Affiliations:** Key Laboratory of Mariculture, Ocean University of China, Ministry of Education, Qingdao 266003, China; wly4137@stu.ouc.edu.cn (L.-Y.W.); tianyuan6350@stu.ouc.edu.cn (Y.T.); wenhaishen@ouc.edu.cn (H.-S.W.); yupeng4489@163.com (P.Y.); yangliu@ysfri.ac.cn (Y.L.); qx@ouc.edu.cn (X.Q.); m17806261553@163.com (Z.-C.G.); zkq@ouc.edu.cn (K.-Q.Z.)

**Keywords:** spotted sea bass, solute carrier 4 family, evolution, gene expression, stress

## Abstract

The solute carrier 4 (SLC4) family is a class of cell membranes transporters involved in base transport that plays crucial roles in diverse physiological processes. In our study, 15 *slc4* genes were identified and annotated in spotted sea bass, including five members of Cl^−^/HCO_3_^−^ exchangers, eight genes coding Na^+^-dependent HCO_3_^−^ transporters, and two copies of Na^+^-coupled borate transporters. The gene sequence and structure, chromosomal and syntenic arrangement, phylogenetic and evolution profiles were analyzed. Results showed that the *slc4* gene in teleosts obviously expanded compared with higher vertebrates, arising from teleost-specific whole genome duplication event. Most gene sites of *slc4* in spotted sea bass were under strong purifying selection during evolution, while positive selection sites were only detected in *slc4a1b*, *slc4a8,* and *slc4a10b*. Additionally, qRT-PCR results showed that different *slc4* genes exhibited distinct branchial expression patterns after alkalinity and salinity stresses, of which the strongly responsive members may play essential roles during these physiological processes. Our study provides the systemic overview of the *slc4* gene family in spotted sea bass and enables a better understanding for the evolution of this family and further deciphering the biological roles in maintaining ion and acid–base homeostasis in teleosts.

## 1. Introduction

The solute carrier 4 (SLC4) family members are integral membrane proteins which carry bicarbonate (HCO_3_^−^) or/and carbonate (CO_3_^2−^) and other electrolytes (e.g., Na^+^ and Cl^−^) across the plasma membrane [1]. These transporters are responsible for maintaining both intracellular and extracellular pH in all eukaryotes [2,3]. Based on the specific transport modes, SLC4 gene family proteins were classified into three functional groups in mammals: (1) Na^+^-independent, Cl^−^/HCO_3_^−^ exchangers including SLC4A1, SLC4A2, and SLC4A3; (2) Na^+^-dependent HCO_3_^−^ transporters, containing electrogenic Na^+^/HCO_3_^−^ cotransporters (SLC4A4, SLC4A5), electroneutral Na^+^/HCO_3_^−^ cotransporters (SLC4A7, SLC4A10), and electroneutral Na^+^-driven Cl^−^/HCO_3_^−^ exchanger (SLC4A8); (3) Na^+^-coupled borate transporter that do not transport bicarbonate, which include the unique member SLC4A11 [2,4,5]. However, the classification of SLC4A9 remains unclear because its function is still incompletely characterized, although both Cl^−^/HCO_3_^−^ exchange and Na^+^-dependent HCO_3_^−^ transport functions were reported in the previous studies about SLC4A9 [6,7,8].

As a result of the importance in regulating pH and maintaining acid–base homeostasis, SLC4 genes have been studied extensively to define their roles in mammals [9,10,11]. Compared with terrestrial vertebrates, studies about *slc4* genes in fishes remain limited, however, accumulating evidences support the hypothesis that *slc4* genes are closely associated with both acid/base and ion regulation in fishes [12,13]. For instance, *slc4a1* and *slc4a4* have been proposed to play critical roles in ion regulatory pathways in gills of zebrafish (*Danio rerio*) [14]. *slc4a1*, *slc4a2*, and *slc4a4* were considered as the contributors for alkali-adaption in gill and kidney of Amur ide (*Leuciscus waleckii*) [15]. Salinity-dependent expression patterns of *slc4a4* genes were observed in the anterior intestine of sea bream (*Sparus aurata* L.) [16]. Similarly, *slc4a4* gene were induced significantly in the intestine of mefugu (*Takifugu obscurus*) [17] and tilapia (*Oreochromis mossambicus*) [13] during seawater-acclimated processes. However, those studies in fishes were focused on single or partial members of *slc4* genes. Therefore, it is of great significance to systematically characterize *slc4* genes and distinguish their functions in teleost.

Spotted sea bass (*Lateolabrax maculatus*) is one of the most promising aquaculture marine fish species in East Asia for its high nutritive value and pleasant taste [18,19]. In addition, spotted sea bass is well known for its ability to adapt to a wide range of salinity environments from complete fresh water (0‰, FW) to sea water (45‰, SW) [20]. Moreover, our previously experiment has proved that spotted sea bass can adapt well to the alkaline water (10 mmol/L) for more than two months and could survive in the water with relative highly alkalinity (18 mmol/L) for more than two weeks. Hence, spotted sea bass represented an ideal model to study the mechanisms of adaptation to extreme salinity and high alkalinity environments. To investigate the potential functions of *slc4* gene family members in response to salinity adaptation and alkalinity stress, in the present study, a complete set of 15 *slc4* genes were identified and characterized in the spotted sea bass. Phylogenetic and syntenic analyses were conducted to confirm the gene annotation and investigate the evolution relationships of *slc4* genes. The gene structure and selective pressure analysis was performed to explore further molecular basis for gene functional studies. Moreover, expression patterns of *L. maculatus slc4* genes were determined in gills during acclimation to extreme salinities (sea water and fresh water, respectively) and high alkalinity environments. Our findings provide insight into the structure, evolution, and potential function of the spotted sea bass *slc4* genes.

## 2. Materials and methods

### 2.1. Ethics Statement

All animal experiments were conducted in accordance with the guidelines and approval of the respective Animal Research and Ethics Committees of Ocean University of China (Permit Number: 20141201). The field studies did not involve endangered or protected species.

### 2.2. Genome-Wide Identification of Spotted Sea Bass Slc4 Genes

The amino acid sequence of *slc4* genes in humans (*Homo sapiens*) and zebrafish (*Danio rerio*) were downloaded from the NCBI database and used as queries for BLAST program. TBLASTN (1 × 10^−^^5^) program was performed to obtain the cDNA sequences by searching the reference genome (PRJNA408177), Iso-Seq (PRJNA515783), and RNA-Seq databases (PRJNA347604) of spotted sea bass. Open reading frames (ORF) of candidate spotted sea bass *slc4* genes were predicted by ORF finder program. The annotation results were further verified by BLAST against the NCBI non-redundant (NR) protein sequence database.

### 2.3. Phylogenetic and Syntenic Analysis

Phylogenetic analysis was conducted by using predicted amino acid sequences of spotted sea bass *slc4* genes combined with the amino acid sequences from several selected vertebrate species, including human (*Homo sapiens*), mouse (*Mus musculus*), chicken (*Gallus gallus*), cow (*Bos taurus*), Japanese medaka (*Oryzias latipes*), Nile tilapia (*Oreochromis niloticus*), torafugu (*Takifugu rubripes*), channel catfish (*Ictalurus punctatus*), Atlantic cod (*Gadus morhua*), European sea bass (*Dicentrarchus labrax*), large yellow croaker (*Larimichthys crocea*), and zebrafish (*Danio rerio*). The phylogenetic tree was constructed using MEGA 7.0 software based on the Neighbor-Joining method and Jones-Taylor-Thornton (JTT) model with bootstrap value setting as 1000.

Syntenic analysis was conducted by comparing genomic regions that harbor *slc4* genes in spotted sea bass with that in zebrafish and tilapia. The genomic regions surrounding *slc4* genes in zebrafish and tilapia were determined according to Ensembl and Genomics databases. Gene full names are presented in Table S1. The spotted sea bass genome database was used to obtain information about chromosome collinear block. The chromosomal location of each *slc4* gene was displayed according to its coordinates on the genome.

### 2.4. Gene Structure and Domain Analysis

The functional domain architectures of *slc4* genes in spotted sea bass were predicted by the SMART 7.0 program. The exon–intron structures of *slc4* genes in spotted sea bass were obtained from the General feature format (GFF) file of reference genome, which were subsequently visualized using online GSDS 2.0 software. The molecular weight (MW, kDa) and isoelectric point (pI) of each putative Slc4 protein were calculated using the ExPASy Prot-Param tool.

### 2.5. Selective Pressure Analysis

The coding sequences of *slc4* genes in human, mouse, chicken, cow, zebrafish, channel catfish, Atlantic cod, Japanese medaka, torafugu, larger yellow croaker, European sea bass, and spotted sea bass were translated into protein sequences and aligned with MEGA7 software, respectively [21], and the aligned protein sequences of each *slc4* gene were used to construct nine unrooted phylogenetic trees (Figure S1). Additionally, the aligned protein sequences were also back-translated into nucleotide-coding sequences for the following selection pressure analysis. To examine whether *slc4* genes of spotted sea bass underwent adaptive sequence evolution, branch-site models were used to test the selection pressure using codeml software of PAML program based on the phylogenetic trees. In the branch site model (model = 2, Nsites = 2), the neutral model constrains a class of sites to have *ω* = 1 (fix_omega = 1, omega = 1), and the selection model allows a class of codons on the foreground branch to have *ω* > 1 (fix_omega = 0, omega = 1.5) [22]. A likelihood ratio test (LRT) was calculated using EasyCodeML (v1.2) for statistical significance [23]. The sites under positive selection were identified based on Bayes methods. The distribution of the positive selection site of the *slc4a1b*, *slc4a8*, and *slc4a10b* of spotted sea bass were shown in the secondary structure by Protter.

### 2.6. Carbonate Alkalinity Exposure Experiment

Carbonate alkalinity solution was prepared using NaHCO_3_ (12.8 mmol/L) and Na_2_CO_3_ (2.6 mmol/L), and fresh water (pH: 7.8 ± 0.4). The carbonate alkalinity (mmol/L) was monitored every day throughout the exposure period with acidimetric titrations. Before the experiment, 45 spotted sea bass (body weight: 140.32 ± 2.56 g) were acclimated in fresh water for 30 days. After that, the experimental fish were randomly assigned into three 100 L square tanks filled with alkaline water (carbonate alkalinity = 18 mmol/L), and the dissolved oxygen concentration, temperature, and pH in each test solution were maintained at 7.1 ± 0.4 mg/L, 22 ± 1 °C and 9.0 ± 0.2, respectively. The fish were not fed during the stress experiment. Three fish individuals in each tank were quickly anesthetized with MS-222 (200 mg/L) and sampled immediately for gill tissues at 0 h, 12 h, 24 h, 48 h, and 72 h after exposure. Samples were immediately placed in liquid nitrogen and then stored at −80 °C for subsequent RNA extraction.

### 2.7. Freshwater and Seawater Transition Experiments

All fish samples were obtained at Shuangying Aquatic Seedling Co., Ltd. (Lijin, Dongying, China). A total of 90 spotted sea bass adults (body weight: 120.66 ± 13.05 g) were randomly selected and equally placed in fresh water (0‰, FW) and sea water (30‰, SW) environments in triplicate tanks at a density of 15 individuals each tank for three weeks before the experiments. After acclimation, individuals in FW were directly transferred to SW which was labeled as FW-SW group. On the contrary, fish that were moved from SW to FW environment were marked as SW-FW group. During the whole experiments process, other experiment conditions were kept constant, including temperature (15 ± 0.5 °C), dissolved oxygen (7.1 ± 0.4 mg/L) and pH (8.0 ± 0.3). For both salinity treatment groups, 3 individuals per tank were anesthetized with MS-222 and sampled for gill tissues at 0 h (before transferring) and 12 h, 1 d, 3 d, and 7 d after transfer.

### 2.8. RNA Extraction and Quantitative Real-Time PCR (qPCR) Analysis

Total RNA was extracted using TRIzol^®^ reagent (Invitrogen, Carlsbad, CA, USA). The concentration and integrity of RNA were examined by using the Biodropsis BD-1000 nucleic acid analyzer (OSTC, Beijing, China) and electrophoresis. Genomic DNA was eliminated and cDNA was synthetized using the PrimeScript™ RT reagent Kit with *g* DNA Eraser (Takara, Otsu, Japan). The cDNA samples were subsequently used as template for following qPCR experiment of *slc4* genes experiments. qPCR reactions were performed on StepOne Plus Real-Time PCR system (Applied Biosystems, Foster City, CA, USA) using SYBR^®^ Premix Ex Taq™ II (Tli RNaseH Plus) (Takara, Otsu, Japan) according to manufacturer instructions. All gene-specific primers used in qPCR were designed using Primer 5 software (Table S2). 18S ribosomal RNA was used as the internal control [24] and the samples were repeated in triplicate technical repeats. The total volume of the reaction is 20 containing 2 μL template cDNA, 0.4 μL each forward/reverse primers, 10 μL SYBR^®^FAST qPCR Master Mix (2×), 0.4 μL ROX, and 6.8 μL of nuclease-free water. qPCR process was designed as follows: 95 °C for 30 s, followed by 40 cycles at 95 °C for 5 s and 58 °C for 30 s. The relative expression level was calculated by 2^−^^∆∆Ct^ method.

### 2.9. Statistical Analysis

Data are expressed as means ± SD. The statistical analysis was performed using SPSS 21 software (SPSS Inc., Chicago, IL, USA). The mean relative mRNA expression levels of *slc4* genes were assessed by one-way ANOVA and Least Significant Difference (LSD) multiple comparison. The significant level was set as *p* < 0.05.

## 3. Results

### 3.1. Characterizations of Slc4 Gene Sequences in Spotted Sea Bass

A total of 15 *slc4* genes were identified in spotted sea bass and further classified into three classes based on the reported classification of Liu and Alper [3,4], including 5 genes of Na^+^-independent Cl^−^/HCO_3_^−^ exchangers (Cl^−^/HCO_3_^−^ exchanger) (*slc4a1a*, *slc4a1b*, *slc4a2a*, *slc4a2b*, *slc4a3*), 8 members of Na^+^-dependent HCO_3_^−^ transporters (Na^+^/HCO_3_^−^ cotransporter) (*slc4a4*, *slc4a5a*, *slc4a5b*, *slc4a7a*, *slc4a7b*, *slc4a8*, *slc4a10a*, *slc4a10b*), and 2 genes belonging to Na^+^-coupled borate transporter (Na^+^/Borate cotransporter) (*slc4a11a* and *slc4a11b*). The predicted amino acids sizes of *slc4* genes ranged from 771 to 1340, with the molecular weights (MWs) were varied from 94.05 kDa to 149.04 kDa. The predicted pI varied from 5.31 to 8.68 (Table 1). The cDNA sequences of these *slc4* genes had been submitted to GeneBank database and their access numbers were listed in Table 1.

### 3.2. Phylogenetic, Syntenic, and Chromosomal Distribution Analysis of slc4 Genes

**Figure 1:**
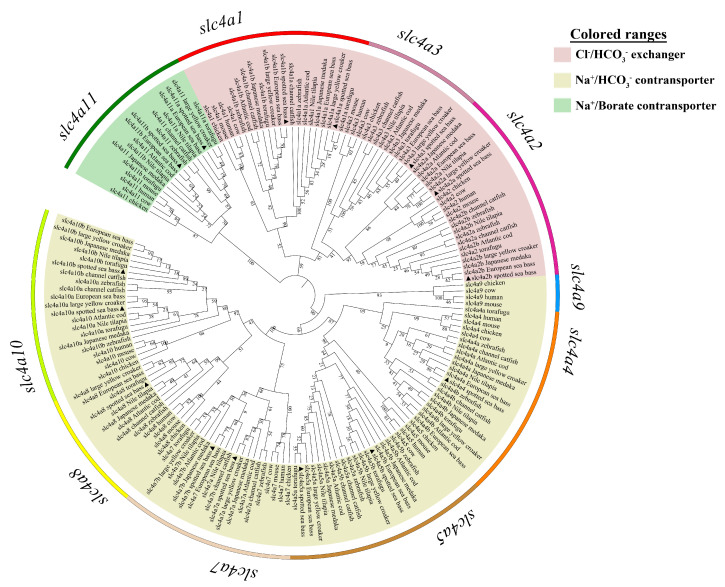
Phylogenetic relationships of *slc4* genes in spotted sea bass and selected vertebrate species. The phylogenetic tree was constructed using MEGA 7.0 software based on the Neighbor-Joining method and Jones-Taylor-Thornton (JTT) model with 1000 replicates. This tree was mainly classified into three classes, including Cl^−^/HCO_3_^−^ exchangers, Na^+^/HCO_3_^−^ cotransporter, and Na^+^/Borate cotransporter.

To confirm the annotation of *slc4* genes in spotted sea bass, a phylogenetic tree was constructed using the protein sequences of *slc4* genes from the selected higher vertebrates and teleosts (Figure 1). According to the topology of phylogenetic tree, these *slc4* genes were divided into ten separate clades and all members of the *slc4* genes in spotted sea bass were cluster together with the expected clades supported by strong bootstrap value. The ten clades were grouped into three major classes, which are consistent with their classification as defined in previous studies [4,25].

**Figure 2:**
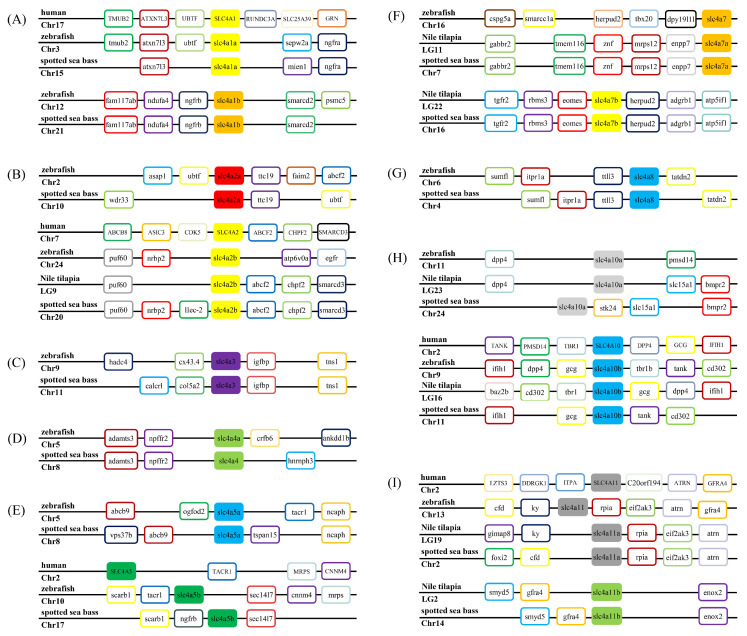
Syntenic analysis of *slc4* genes in zebrafish, Nile tilapia, and spotted sea bass. (**A**–**I**): *slc4a1*–*slc4a11*. Full gene names are provided in Table S2.

The annotation of three *slc4a3*, *slc4a4*, and *slc4a8* genes with single copy could be well supported by the phylogenetic relationships (Figure 1). To provide additional evidence for the annotation and evaluate the evolution relationships of *slc4* genes with multiple gene copies (*slc4a1*, *slc4a2*, *slc4a5*, *slc4a7*, *slc4a10*, and *slc4a11*), syntenic analysis were conducted to compare the neighbored genetic regions of *slc4* genes among zebrafish, Nile tilapia, and spotted sea bass (Figure 2). The results indicated that *slc4* genes in spotted sea bass shared a conserved genomic neighborhood with zebrafish and Nile tilapia, confirming the annotation of the duplicated *slc4* gene. Therefore, our results not only well support the annotation of *slc4* genes in spotted sea bass, but also indicate the *slc4* genes were highly conserved in evolution.

**Figure 3:**
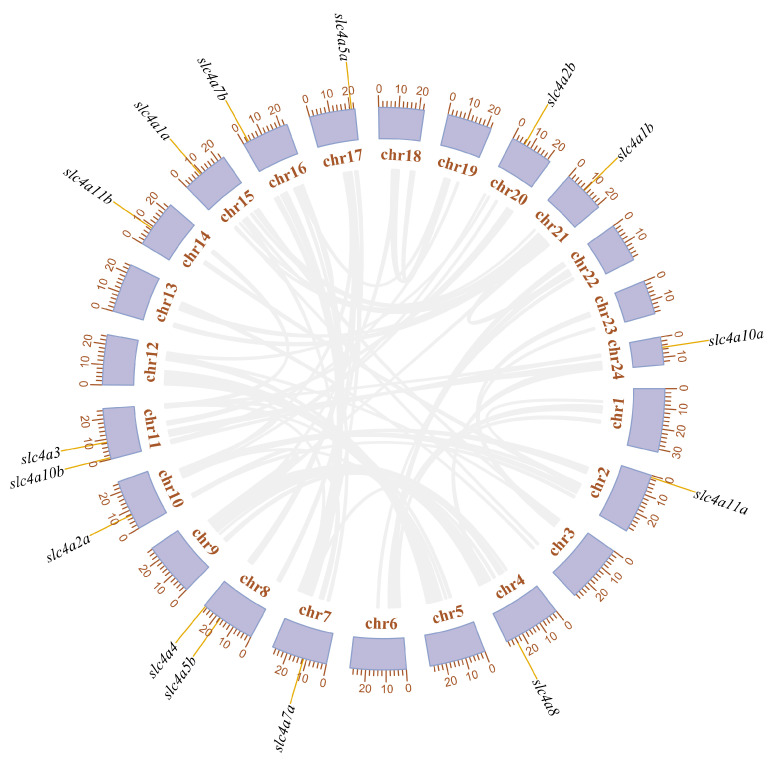
Chromosomal locations and duplication modes among the *slc4* genes of spotted sea bass. Genomic synteny is displayed in gray lines.

In addition, *slc4* genes in spotted sea bass were dispersed among 13 chromosomes (chrs), including chr2, chr4, chr7, chr8, chr10, chr11, chr14, chr15, chr16, chr17, chr20, chr21, and chr24 (Figure 3). Two *slc4* genes (*slc4a4* and *slc4a5b*) were located on chr8 and two (*slc4a3* and *slc4a10b*) were located on chr11, and others were distributed on different chromosomes. Therefore, according to the chromosomal synteny analyses and location information of each *slc4* genes, we were able to speculate that duplicated *slc4* genes of spotted sea bass were mainly generated due to the whole genome duplication (WGD) event.

### 3.3. Gene Structure Analysis of Spotted Sea Bass slc4 Genes

**Figure 4:**
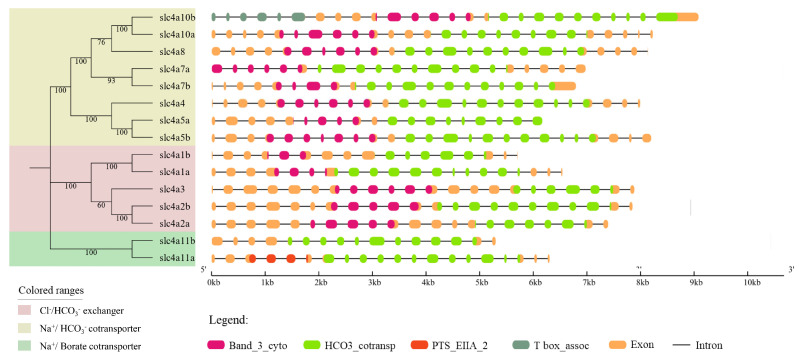
Schematic representation of gene structures of *slc4* genes in spotted sea bass. The phylogenetic relationship was shown on the left. The boxes represented exons and lines were intron. The functional domains were marked with different colors.

To investigate the structural diversities of *slc4* genes of spotted sea bass, their exon–intron structures were compared (Figure 4). Results showed that most members of Cl^−^/HCO_3_^−^ exchanger possessed 20–22 exons, except for *slc4a1b* with 17 exons. Among Na^+^/HCO_3_^−^ group, the gene structures of *slc4a4*, *slc4a5b*, *slc4a8*, *slc4a10a*, and *slc4a10b* were comprise of 25 or 26 exons, while 19–21 exons were observed in *slc4a5a*, *slc4a7a*, and *slc4a7b*, respectively. In addition, the Na^+^/Borate group members, *slc4a11a* and *slc4a11b* contained 20 and 16 exons, respectively. In general, the exon–intron organizations were mainly consistent with the phylogenetic clustering results. Moreover, four functional domains (Band_3_cyto, HCO3_cotransp, T box_assoc, and PTS_EIIA_2) were predicted in *slc4* genes (Figure 4). The Band_3_cyto and HCO3_cotransp domains presented in all *slc4* genes of spotted sea bass except *slc4a11a* and *slc4a11b*, whereas T box_assoc domain was only observed in *slc4a10b* and PTS_EIIA_2 was presented solely in *slc4a11a* (Figure 4).

### 3.4. Copy Number Comparison of slc4 Genes in Selected Vertebrates

**Figure 5:**
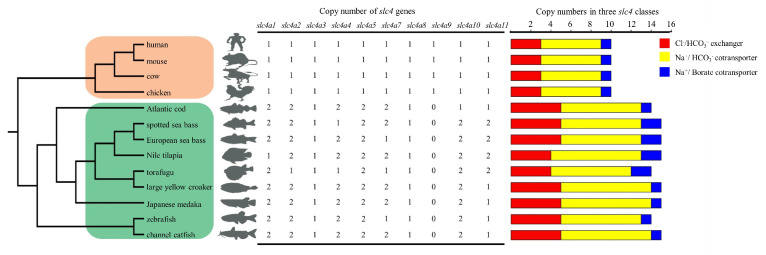
Copy numbers of *slc4* genes in 13 representative vertebrates. Branch shades represent the different groups: higher vertebrates were marked by orange shade and teleosts were shown in green. The copy number of three *slc4* classes were represented by colored rectangle: Cl^−^/HCO_3_^−^ exchangers in red, Na^+^/HCO_3_^−^ transporters in yellow and Na^+^/Borate transporters in blue.

The copy number of *slc4* genes from 13 representative species, covering species from higher vertebrates (human, mouse, cow and chicken) to teleosts (Atlantic cod, Japanese medaka, Nile tilapia, torafugu, channel catfish, large yellow croaker, European sea bass, spotted sea bass and zebrafish) were exhibited in Figure 5. In general, the number of *slc4* genes in the selected vertebrates varied from 10 to 15. Notably, all *slc4* genes in the tested higher vertebrates harbored single copy, while duplicated *slc4* gene copies were frequently found in teleost genome, including *slc4a1*, *slc4a2*, *slc4a4*, *slc4a5*, *slc4a7*, *slc4a10*, and *slc4a11* (Figure 5). This result indicated that *slc4* gene family was significantly expanded in teleosts during evolution. In addition, it was observed that *slc4a9* gene was absent in all examined teleost species (Figure 5).

### 3.5. Selective Pressure Analysis

**Figure 6:**
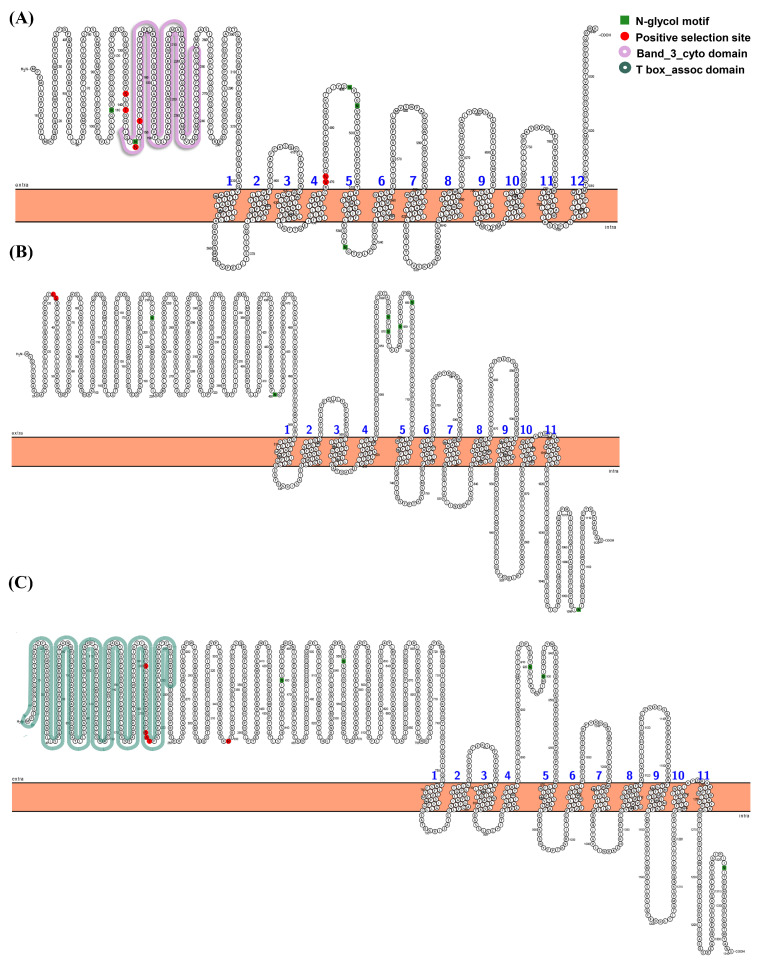
Distribution of positive selection sites of the *slc4* genes of spotted sea bass predicted by the branch-site models. Positively selected sites (*p* > 95%) in the predicted secondary structures of *slc4a1b* (**A**), *slc4a8* (**B**), and *slc4a10b* (**C**) were labeled in red. The functional domains were marked with different colors.

Using branch-site models, null and alternative models were compared to determine whether *slc4* genes underwent positive selection in the particular lineages. The detailed parameter estimates, including likelihood value, LRT tests, *p*-values, and positive selected sites were summarized in Table 2. The LRT tests indicated that alternative model fitted the data significantly better than null model in *slc4a1b*, *slc4a8*, and *slc4a10b* of spotted sea bass (Table 2). There were 6 (138S, 141T, 148N, 152T, 470V, 471N), 2 (33L, 34S), and 5 (200S, 214S, 215A, 216T, 349R) sites were found that likely to be under positive selection (*p* < 0.05) along *slc4a1b*, *slc4a8*, and *slc4a10b*, respectively. No significant positive selection site was found in other spotted sea bass *slc4* genes, which implied purifying selection during evolution. In addition, the majority of the positively selected sites we found were located in the N-terminal extracellular region. According to the position of *slc4a1b*, 2 positively sites (148N, 152T) were located on the Band_3_cyto domain, while 4 positively sites (200S, 214S, 215A, 216T) were located on T-box assoc domain according to the position of *slc4a10b* (Figure 6). It suggested that the functional diversification and adaptation might occurred in the three *slc4* genes in spotted sea bass.

### 3.6. Expression Patterns of slc4 Genes in Response to Alkalinity Stress

**Figure 7:**
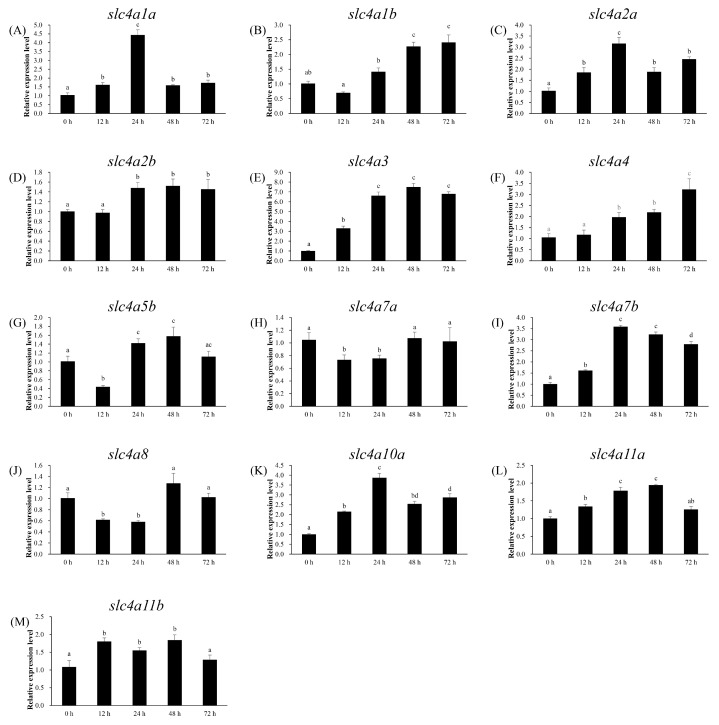
Expression of *slc4* genes in gill of spotted sea bass after alkalinity stress. (**A**–**M**): *slc4a1a*–*slc4a11b*. Significant differences (*p* < 0.05) were indicated with different letters.

The expression profile of *slc4* genes in gill of spotted sea bass was determined by qPCR at different time points after alkalinity stress. The results showed that 13 *slc4* genes (*slc4a5a* and *slc4a10b* were not detected in gill) were expressed differentially in the gill with distinct patterns (Figure 7). Among them, the expression of *slc4a3* showed the strongest and most immediately up-regulated trend, with the relative mRNA levels at 12 h triple that at 0 h, and reached to a higher expression level at 24 h (Figure 7E). The highly expression was maintained at 48 h and 72 h with more than a 6-fold change compared with its expression at 0 h point (Figure 7E). The expressions of *slc4a1a*, *slc4a2a*, *slc4a7b*, and *slc4a10a* exhibited a similar pattern that their mRNA expressions were induced slightly at 12 h, and then up-regulated significantly at 24 h (> 3-fold), but decreased after 48 h (Figure 7A,C,I,K). The expression of *slc4a1b* and *slc4a4* showed a continuous up-regulation trend after 24 h, and reached to their peak at 72 h with expression values 2.4- and 3.2-fold compared with 0 h (Figure 7B,F). The expression of *slc4a5b* showed a trend of decline at the 12 h (2.3-fold) and then rise slightly at the 24 h compared with its expression at 0 h (Figure 7G). The expressions of the remaining *slc4* genes including *slc4a2b*, *slc4a7a*, *slc4a8*, *slc4a11a*, and *slc4a11b* showed slight variation (< 2-fold) during the entire experiment (Figure 7D,H,J,L,M).

### 3.7. Branchial Expression Patterns of slc4 Genes During FW and SW Acclimation

**Figure 8:**
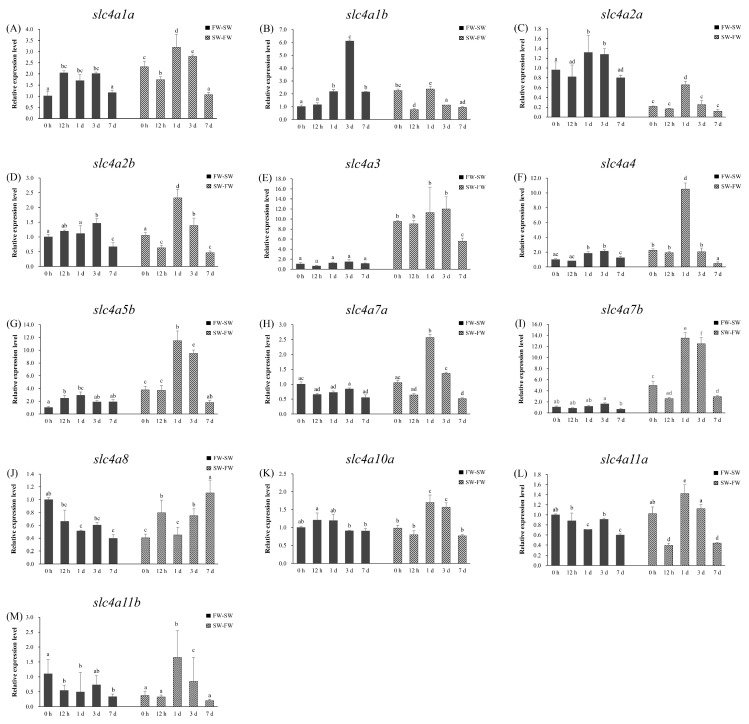
Expression of *slc4* genes in gill of spotted sea bass after freshwater-saltwater (FW-SW) and SW-FW transfer. (**A**–**M**): *slc4a1a*–*slc4a11b*. Fish were transferred from FW to SW or SW to FW as control at 0 h. Significant differences (*p* < 0.05) were indicated with different letters.

In order to investigate the potential function of *slc4* genes in spotted sea bass adapted to fresh water and sea water, the mRNA expression patterns of 13 *slc4* genes with remarkable expression levels in the gill were determined using qPCR. As shown in Figure 8, after transferred from FW to SW (FW-SW), 12 of 13 *slc4* genes (except *slc4a3*, Figure 8E) were found to be differentially expressed at varying degrees (*p* < 0.05). The expressions of 8 out of 13 *slc4* genes (*slc4a1a*, *slc4a1b*, *slc4a2a*, *slc4a2b*, *slc4a4*, *slc4a5b*, *slc4a7b*, and *slc4a10a*) were elevated firstly and then decreased with the time, whereas most of them did not change much (< 2-fold) except *slc4a1a*, *slc4a1b*, and *slc4a5b* (Figure 8A–D,F,G,I,K). The expression levels of *slc4a1b* were up-regulated two-fold at 1 d, and highly increased at 3 d (> 6-fold) compared with its lowest expression level at 0 h, then decreased by 7 d (Figure 8B). Similarly, the expression of *slc4a5b* was induced significantly from 12 h (2.5-fold) and then declined after 3 d (Figure 8G). On the contrary, the rest *slc4* genes, especially *slc4a8*, *slc4a11a*, and *slc4a11b*, the expressions of which were suppressed significantly compared with that at 0 h following transfer from FW to SW (Figure 8J,L,M).

As shown in Figure 8, all of 13 *slc4* genes were found to be differentially expressed at varying degrees during FW acclimation processes. In fish transferred from SW-FW, the branchial mRNA expression of most *slc4* genes decreased (*slc4a1a*, *slc4a2a*, *slc4a2b*, *slc4a4*, *slc4a7a*, *slc4a7b*, *slc4a10a*, and *slc4a11a*) or remained constant (*slc4a3*, *slc4a5b*, *slc4a11b*) at 12 h, and then significantly induced and reached the highest expression value by day 1 (Figure 8). Among these genes, the biggest up-regulation level was observed in *slc4a4*, *slc4a5b*, and *slc4a7b*, with the expressions 4.7-, 3.0- and 2.7-fold higher compared with its expression at 0 h (Figure 8F,G,I). The expressions of almost all *slc4* genes decreased by 3 d, with only one exception that the branchial mRNA level of *slc4a8* kept increasing until 7 d after transferred from SW to FW (Figure 8J). Notably, the relative expression levels of *slc4a2a* in the gill was significant higher (4.5-fold) in seawater- than freshwater-acclimated fish during the entire experimental process, by contrast, the *slc4a3* showed the opposite pattern that the overall mRNA was more abundant (9.2-fold) in freshwater-acclimated fish (Figure 8C,E).

## 4. Discussion

Slc4 family proteins have been studied extensively in mammals as bicarbonate transporters, responsible for diverse physiological processes including cellular pH regulation, cell volume mediation, and NaCl absorption [4,25,26]. However, the *slc4* genes and their physiological roles have not been well elucidated in teleosts. To fill the gap, in the present study, a comprehensive analysis of *slc4* gene family was performed in spotted sea bass.

### 4.1. Features of slc4 Genes in Spotted Sea Bass

A complete set of 15 *slc4* genes were identified from genomic and transcriptomic databases of spotted sea bass. It has been well documented that *slc4* genes are generally classified into three clades based on their known transport function [2,4]. In the present study, 15 *slc4* genes of spotted sea bass were comprised of 5 Cl^−^/HCO_3_^−^ exchangers (*slc4a1a*, *slc4a1b*, *slc4a2a*, *slc4a2b*, *slc4a3*), 8 Na^+^-dependent HCO_3_^−^ transporters (*slc4a4*, *slc4a5a*, *slc4a5b*, *slc4a7a*, *slc4a7b*, *slc4a8*, *slc4a10a*, *slc4a10b*) and 2 Na^+^-coupled borate transporters (*slc4a11a* and *slc4a11b*). Phylogenetic and syntenic analysis provided additional evidence for the annotation of *slc4* genes and gain insight into their evolutionary relationships. The phylogenetic tree was mainly divided into three major clades consistent with the function classifications of *slc4* genes in human (Figure 1) [2]. The orthologous *slc4* genes from tested species were clustered together as expected (Figure 1), and the similar neighboring genes of *slc4* genes were comprised of zebrafish, tilapia and spotted sea bass (Figure 2), which not only confirm the gene annotation, but also demonstrate the relative conservation of *slc4* genes in evolution.

Gene structure analysis of *slc4* genes in spotted sea bass showed their exon–intron and functional domain patterns (Figure 4). HCO3_cotransp functional domains were located at the C terminal of all the *slc4* genes in spotted sea bass, which were supposed to be responsible for their anion transport (GO:0006820). Band_3_cyto domains in both Cl^−^/HCO_3_^−^ exchangers and Na^+^-dependent HCO_3_^−^ transporters were primarily considered as an anchoring site for other membrane-associated proteins such as ankyrin and aldolase [27]. Overall, members from the same classification with conserved exon–intron and functional domain patterns further support the functional similarities among *slc4* gene members in the same evolutionary clade.

The number of *slc4* genes differed between higher vertebrates and teleosts, due to the duplicated *slc4* genes were common founded in teleost, such as *slc4a1*, *slc4a2*, *slc4a4*, *slc4a5*, *slc4a7*, *slc4a10*, and *slc4a11*. Notably, these duplicated *slc4* genes were distributed on different chromosomes instead of tandem duplication arrangement (Figure 3). Therefore, it was reasonable to assume that these duplicated *slc4* genes were derived from teleost-specific WGD event [28,29]. Additionally, the absence of *slc4a9* in teleosts may be resulted from gene losses during evolution [30,31]. Additionally, previous studies have shown that positive selection, or adaptive sequence evolution, is considered as the hallmark of evolutionary change and molecular adaptation [22,32]. To better understand the evolutionary dynamics of *slc4* genes in spotted sea bass, positive selection analysis was conducted using branch-site model. Our results showed that most sites of spotted sea bass *slc4* genes were under purifying selection during evolution (Table 2), which is the expected outcome in evolutionary studies that strong purifying selection is used to keep the protein functional integrity [33]. Positive selection sites were identified in *slc4a1b* (6 sites), *slc4a8* (2 sites), and *slc4a10b* (5 sites) (Table 2). Two sites of *slc4a1b* under selective pressures were located in the known protein functional domain—Band_3_cyto—which suggested that positive selection may have important roles in driving changes in the binding capability in spotted sea bass [27].

### 4.2. Function Studies of slc4 Genes of Spotted Sea Bass in Response to Salinity and Alkalinity Stresses

Compared with higher vertebrates, function study of *slc4* genes in fish species lags far behind. In euryhaline teleosts, gill is the essential organ responsible for ionic regulation and acid–base regulation [34]. Hence, to investigate the potential roles of *slc4* genes in adaptation to extreme salinity and high alkalinity environment, the branchial expression patterns of *slc4* family genes were determined by qPCR in spotted sea bass after freshwater and seawater transfer, and alkalinity stress.

#### 4.2.1. Anion Exchanger Proteins (AEs)

This group includes at least three anion exchanger proteins (AE1, AE2, AE3) in mammals, encoded by SLC4A1, SLC4A2, and SLC4A3, respectively. Five AEs encoding genes (*slc4a1a*, *slc4a1b*, *slc4a2a*, *slc4a2b*, *slc4a3*) of spotted sea bass were identified in our study (Table 1). In accordance with the gene structure of mammalian AEs, the predicted cytoplasmic N terminus of spotted sea bass *slc4a2* (*slc4a2a*, *slc4a2b*) and *slc4a3* are longer than that of *slc4a1* (*slc4a1a*, *slc4a1b*) (Figure 4). AE anion exchangers have been widely reported for their roles in mediating the electroneutral exchange of one monovalent anion for another (Cl^−^ and HCO_3_^−^ are the preferred substrates) across the plasma membrane in mammals [2], and their functions are reviewed in details by Alper [4]. By contrast, the in-depth study about the ion- and acid-base regulation function of AEs in teleosts is still lacking. In this study, the mRNA expressions of anion exchanger members in spotted sea bass were sensitive to changes in water pH and salinity (Figure 7A–E and Figure 8A–E). In teleosts, AE1 protein was found to present in gill mitochondrion-rich cells (MRCs), which is key sites responsible for both salt and acid regulation. [35,36,37]. In FW fish, AE1 at the apical side of MRCs has been speculated to absorb Cl^−^ and secrete HCO_3_^−^, whereas the AE1 located to the basolateral membrane of acid-secreting MRCs was suggested to be involved in acid secretion instead of Cl^−^ uptake [25]. The branchial expression of zebrafish *slc4a1b* in high Na^+^ environment was lower than that in low Na^+^ environment, while the expression in acidic water was higher than that in fresh water environment [25]. In SW fish, the basolateral AE1 in MRCs showed to play a dual role in acid–base regulation and salt secretion. For example, in SW-acclimated medaka, treatment of inhibitor of AE suppressed both H^+^ and Cl^-^ secretion of MRCs, and both AE1a and AE1b mRNA increased in fish acclimated to acidified SW [35]. In euryhaline spotted green pufferfish (*Tetraodon nigroviridis*), the protein abundance of AE1 in membrane fractions of gills was salinity dependent [37], and later proved that the branchial AE1 interacted with carbonic anhydrase II (CAII) in the membrane of gill Na^+^/K^+^-ATPase-immunoreactive cells to complete Cl^−^/HCO_3_^−^ transport. [37]. For *slc4a2*, the functional experiment has proved that the anion exchanger activity in zebrafish was inhibited by both intracellular and extracellular acidic pH and activated by alkaline pH [12], which corresponded to the mRNA variation pattern in our study (Figure 7C,D and Figure 8C,D). In spotted sea bass, after alkalinity challenge, the most dramatic change of branchial mRNA was observed for *slc4a3* that was induced more than 6-fold compared with its expression at 0 h (Figure 7E). The study about the functions of *slc4a3* in teleost was limited to zebrafish, that anion exchange activity of AE3 was suppressed at pH of 5.0, but greatly activated at pH 8.5 [38]. Although these findings provided evidences that AEs may play important roles in response to high alkalinity environment and osmoregulation in spotted sea bass and other fishes, however, the physiological roles of AEs-mediated anion exchange in teleost remain to be elucidated.

#### 4.2.2. Na^+^-Coupled Bicarbonate Transporters (NCBTs)

Five mammalian NCBT proteins were identified in SLC4 family, including two electrogenic Na^+^, HCO_3_^−^ cotransporters (NBCe2 and NBCe2), two electroneutral Na^+^, HCO_3_^−^ cotransporters (NBCn1 and NBCn2), and one electroneutral Na^+^-driven Cl^−^, HCO_3_^−^ exchanger (NDCBE). NCBTs are involved in intracellular pH regulation in non-epithelial tissues, and play functions in transcellular movement of acid–base equivalents and controlling intracellular pH for epithelia tissues [39]. However, their functions in teleosts were only partially described. In this study, we identified 8 genes encoding for NCBT proteins in spotted sea bass (Table 1), and their expression levels were detected after alkalinity and salinity change.

Slc4a4 (NBCe1) and Slc4a5 (NBCe2) were electrogenic Na^+^-coupled HCO_3_^−^ transporter and responsible for the accumulation of intracellular HCO_3_^−^ by moving Na^+^ and HCO_3_^−^ into cells [40,41]. The difference is that Slc4a5 transports Na^+^ and HCO_3_^−^ in a ratio of 1:2, while Slc4a4 converts them in a ratio of 1:2, 1:3, or 1:4 [2,41,42]. Our results suggested that *slc4a4* and *slc4a5* genes in spotted sea bass might play important functions in gills for ion- and acid-regulation as their expression levels were remarkable regulated after alkalinity challenge and transferred from SW to FW (Figure 7F,G and Figure 8F,G). In agreement with our finding, the functional involvements of *slc4a4* in the gills were reported in eel (*Anguilla rostrata*) [43]. In addition, as the intestinal HCO_3_^−^ secretion is the key mechanism to enable luminal aggregate formation and water absorption, and transepithelial movement of basolateral HCO_3_^−^ is mediated by *slc4a4* present in the basolateral membrane, several studies have reported the essential function of Slc4a4 in the intestine of marine fishes like European sea bass (*Dicentrarchus labrax*), sea bream (*Sparus aurata* L.), Mozambique tilapia (*Oreochromis mossambicus*) [13,44,45], and hybrid tilapia (*Oreochromis mossambicus* female × *O. urolepis hornorum* male) [46]. In that case, the expression pattern of *slc4a4* needs to be investigated in spotted sea bass in a future study. Moreover, we also detected the highly induced branchial expression of *slc4a5b* in spotted sea bass following transfer from SW to FW, however, the roles of NBCe2 in the regulation of sodium homeostasis and pH balance have been only reported in mammals [47].

NBCn1 encoded by SLC4A7 generally moves 1 Na^+^ and 1 HCO_3_^−^ into the cells, and is widely distributed in both epithelial and nonepithelial tissues [39]. For Na^+^-coupled bicarbonate transporters, the function studies in teleosts was almost in a blank. Our results provide the evidence of the potential involvement of *slc4a7* for ion- and acid–base in gills of spotted sea bass. The mRNA level of *slc4a7b* increased significantly after challenged by high alkalinity (Figure 7I), and the expressions of both *slc4a7a* and *slc4a7b* were induced after transferring from SW to FW, especially *slc4a7b* present more than a 10-fold higher expression level than its in SW environment (Figure 8H,I). In addition, the *slc4a10* gene, which is a basolateral Na^+^ loader sharing the highest amino acid homology with *slc4a7* and *slc4a8* in mammals, showed significantly differentially expressed after alkalinity stress in spotted sea bass (Figure 7K).

#### 4.2.3. Na^+^-Coupled Borate Transporter (NaBC)

Slc4a11 encoding the Na^+^-coupled borate cotransporter (NaBC1), is the only *slc4* family member that does not transport HCO_3_^−^. Slc4a11 was reported as an electrogenic, voltage-regulated, Na^+^-coupled B(OH)_4_ transporter in the presence of borate, while in the absence of borate, Slc4a11 was speculated to conduct Na^+^ and OH^−^(H^+^) transportation [48]. Recently, Slc4a11 was identified as a novel NH_3_/H^+^ co-transporter with an apparent stoichiometry of 1:2 NH_3_/H^+^ [49]. Our results indicated Slc4a11 of spotted sea bass may involve in the regulation of ion- and acid–base/nitrogen homeostasis (Figure 7L,M and Figure 8L,M), although few related cases have been reported in the teleosts.

It was worth noting that whether these *slc4* genes of spotted sea bass up- or down-regulated by environmental salinity, most of their expressions returned to nearing the initial level at 7 d in hyperosmotic and hypoosmotic transition experiment. It may indicate that as euryhaline species, spotted sea bass could back to homeostasis when acclimated to FW or SW within 7 days.

## 5. Conclusions

A genome-wide identification and comprehensive analysis of *slc4* gene family in spotted sea bass was completed in this study. The 15 *slc4* genes were sorted into three groups: Cl^−^/HCO_3_^−^ exchangers, Na^+^-dependent HCO_3_^−^ transporters, and Na^+^-coupled borate transporter. The phylogenetic relationship, sequence structure, and syntenic arrangement supported the annotation and homology of these genes. Most gene sites were under purifying selection during evolution except a few positive selection sites detected in *slc4a1b*, *slc4a8*, and *slc4a10b*. In addition, the branchial gene expression patterns showed that several *slc4* genes of spotted sea bass were differentially expressed at varying degrees in response to alkalinity stress and salinity changes. Among them, *slc4a3* was induced most significantly after alkalinity stress. Following transfer from fresh water to sea water (FW-SW), *slc4a1b* and *slc4a11b* showed the most up-regulated and down-regulated trend, respectively, meanwhile *slc4a4*, *slc4a5b*, and *slc4a7b* exhibited the biggest up-regulation level after transferring from sea water to fresh water (SW-FW). In general, our study provided the fundamental information about the features of *slc4* family in spotted sea bass, which help to better understanding the evolution of this family, and their biological roles in teleost species.

## Figures and Tables

**Table 1:** Summary of characteristics of *slc4* genes in spotted sea bass.

Classification	Gene Name	CDS Length (bp)	Predicted Protein Length (Amino Acid)	Molecular Weight (kDa)	Isoelectric Point (pI)	Accession Number of NCBI
Cl^−^/HCO_3_^−^ exchanger	*slc4a1a*	2766	921	103.29	5.31	MN909728
	*slc4a1b*	2523	840	94.05	8.68	MN909729
	*slc4a2a*	3417	1138	126.82	5.99	MN909730
	*slc4a2b*	3678	1225	137.11	6.09	MN909731
	*slc4a3*	3702	1234	139.71	5.85	MN909732
Na^+^/HCO_3_^−^ cotransporter	*slc4a4*	3213	1070	120.51	6.23	MN909733
	*slc4a5a*	2586	861	96.92	5.80	MN909734
	*slc4a5b*	3423	1140	129.03	6.63	MN909735
	*slc4a7a*	2799	932	103.90	6.82	MN909736
	*slc4a7b*	3015	1004	111.26	6.66	MN909737
	*slc4a8*	3363	1120	125.37	6.03	MN909738
	*slc4a10a*	3261	1086	122.58	6.18	MN909739
	*slc4a10b*	4023	1340	149.04	5.83	MN909740
Na^+^/Borate cotransporter	*slc4a11a*	2529	842	94.91	6.11	MN909741
	*slc4a11b*	2316	771	86.84	6.73	MN909742

**Table 2:** The parameters and statistical significances of likelihood ratio tests for the branch-site models.

Model	np	LnL	Models Compared	2△LnL	df	*p*	Positively Selected Sites
Null model A—*slc4a1a*	43	−27215.89837	MA vs. NMA	0.00000	1	1.00000	
Model A—*slc4a1a*	44	−27215.89837					
Null model A—*slc4a1b*	43	−27204.09798	MA vs. NMA	42.52516	1	0.00000	138S * 141T ** 148N ** 152T ** 470V ** 471N **
Model A—*slc4a1b*	44	−27182.83540					
Null model A—*slc4a2a*	43	−16901.01428	MA vs. NMA	2.0 × 10^−5^	1	0.99643	
Model A—*slc4a2a*	44	−16901.01429					
Null model A—*slc4a2b*	43	−16900.85034	MA vs. NMA	0.00000	1	1.00000	
Model A—*slc4a2b*	44	−16900.85034					
Null model A—*slc4a3*	27	−22702.55961	MA vs. NMA	2.6 × 10^−4^	1	0.4791	
Model A—*slc4a3*	28	−22702.55948					
Null model A—*slc4a4*	43	−27709.75538	MA vs. NMA	0.00000	1	1.00000	
Model A—*slc4a4*	44	−27709.75538					
Null model A—*slc4a5a*	45	−22011.18613	MA vs. NMA	0.08432	1	0.77153	
Model A—*slc4a5a*	46	−22011.14397					
Null model A—*slc4a5b*	45	−22013.51479	MA vs. NMA	0.00000	1	1.00000	
Model A—*slc4a5b*	46	−22013.51479					
Null model A—*slc4a7a*	39	−3957.77188	MA vs. NMA	0.00000	1	1.00000	
Model A—*slc4a7a*	40	−3957.77188					
Null model A—*slc4a7b*	39	−3957.65280	MA vs. NMA	0.23816	1	0.62554	
Model A—*slc4a7b*	40	−3957.77188					
Null model A—*slc4a8*	27	−7554.82350	MA vs. NMA	7.70544	1	0.00551	33L * 34S **
Model A—*slc4a8*	28	−7550.97078					
Null model A—*slc4a10a*	43	−27358.78420	MA vs. NMA	0.28100	1	0.59605	
Model A—*slc4a10a*	44	−27358.64370					
Null model A—*slc4a10b*	43	−27346.85330	MA vs. NMA	31.10466	1	0.00000	200S * 214S ** 215A ** 216T ** 349R *
Model A—*slc4a10b*	44	−27331.30097					
Null model A—*slc4a11a*	35	−6835.90728	MA vs. NMA	0.00000	1	1.00000	
Model A—*slc4a11a*	36	−6835.90728					
Null model A—*slc4a11b*	35	−6835.90728	MA vs. NMA	0.00000	1	1.00000	
Model A—*slc4a11b*	36	−6835.90728					

LnL: The natural logarithm of the likelihood value; np: number of parameters; 2△LnL: twice the difference in LnL between the two models compared; sites inferred to be under positive selection at the 95% level are labeled with single asterisk (*) and those at the 99% level are labeled with two asterisks (**).

## Supplementary Materials

Supplementary Materials can be found at https://www.mdpi.com/2073-4425/11/11/1271/s1. Table S1: List of sequences of primers for real-time PCR. Table S2: Abbreviations of gene names used in synteny analysis. Figure S1: Phylogenetic tree of teleosts *slc4* genes for branch-sites model. The phylogenetic tree was constructed using MEGA 7.0 software based on the Neighbor-Joining method and Jones–Taylor–Thornton (JTT) model with 1000 replicates. The *slc4* genes of spotted sea bass were marked by green color.
